# Northern forest tree populations are physiologically maladapted to drought

**DOI:** 10.1038/s41467-018-07701-0

**Published:** 2018-12-10

**Authors:** Miriam Isaac-Renton, David Montwé, Andreas Hamann, Heinrich Spiecker, Paolo Cherubini, Kerstin Treydte

**Affiliations:** 1 Department of Renewable Resources, University of Alberta, Edmonton, AB T6G 2H1 Canada; 2 Chair of Forest Growth and Dendroecology, Albert-Ludwigs-Universität-Freiburg, Tennenbacherstrasse 4, 79106 Freiburg, Germany; 3 Swiss Federal Institute for Forest, Snow and Landscape Research WSL, Zürcherstrasse 111, CH-8903 Birmensdorf, Switzerland

**Keywords:** Population genetics, Drought, Climate-change impacts, Boreal ecology, Ecophysiology

## Abstract

Northern forests at the leading edge of their distributions may not show increased primary productivity under climate warming, being limited by climatic extremes such as drought. Looking beyond tree growth to underlying physiological mechanisms is fundamental for accurate predictions of forest responses to climate warming and drought stress. Within a 32-year genetic field trial, we analyze relative contributions of xylem plasticity and inferred stomatal response to drought tolerance in regional populations of a widespread conifer. Genetic adaptation leads to varying responses under drought. Trailing-edge tree populations produce fewer tracheids with thicker cell walls, characteristic of drought-tolerance. Stomatal response explains the moderate drought tolerance of tree populations in central areas of the species range. Growth loss of the northern population is linked to low stomatal responsiveness combined with the production of tracheids with thinner cell walls. Forests of the western boreal may therefore lack physiological adaptations necessary to tolerate drier conditions.

## Abstract (web summary)

Northern tree populations may not benefit under climate change, with implications for assisted migration and range expansion. Here, Isaac-Renton et al. show that leading-edge lodgepole pine populations have fewer characteristics of drought-tolerance, so may not adapt to tolerate drier conditions.

## Introduction

Northern forests play a key role in carbon and water cycles, and influence climate forcings and feedbacks^1–3^. Northern latitudes will be disproportionately affected by climate change, and reports of boreal forest decline^4–7^ are beginning to replace earlier optimism of benefits due to warming^8,9^. It is therefore critical to assess the physiological capacity of northern forests to cope with changing climates, including climatic extremes. Drought in particular is anticipated to become more frequent and severe as greater climatic variability coincides with higher evaporative demand^2,10–12^. Although trees have high plasticity, there are limitations to trees’ adaptive capacity, meaning drought may cause substantial forest productivity losses and mortality. Severe drought has been linked to vascular damage and disrupted water columns causing hydraulic failure in trees^13^. Prolonged droughts may also increase forest mortality through slow depletion of tree internal carbon reserves, known as carbon starvation^14^.

Both wood hydraulic traits and stomatal regulation of water loss affect vascular damage and photosynthesis under moisture limitation^15–19^. Combined, xylem morphology and stomatal responsiveness contribute to a continuum of tree physiological behaviors for coping with drought. Drought-avoidant behaviour involves closure of stomata under drier conditions to maintain xylem water potentials, thereby reducing cavitation risk that may lead to hydraulic failure^18,20^. Alternatively, trees may produce more cavitation-resistant xylem through the formation of tracheids with thicker cell walls and smaller lumen diameters. This drought-tolerant behaviour could enable stomata to remain open under prolonged drought, which may help avoid depletion of carbon reserves. While several studies point to differences in drought coping behaviors among forest tree species^14,18^, very few studies have examined differences in water-use behaviours of populations within species^21,22^. More information on the responses within widespread species is needed^19,23^, both in terms of the amplitude of the response and the physiological mechanisms underlying these responses to drought.

Local adaptations in drought physiology are likely: wide-ranging tree species that occur across a range of climate conditions often consist of populations that have developed unique multi-trait polygenic adaptations to local climates^24,25^. Provenance trials, also referred to as common garden trials, provide an experimental setting to study differences in local adaptation and effects of seed transfers^26–28^: by growing multiple seed sources in common gardens, where test environments are homogenous, population-level differences can be revealed. Common garden trials therefore are suitable experiments to test intraspecific variation in water-use strategies^21^. Large reciprocal transplant experiments additionally act as real-world climate change laboratories since transferring northern seeds southward simulates projected warming, while moving southern seeds northward tests survival under assisted migration. Further combining multi-decade provenance trials with tree-ring methodologies facilitates the observation of a population’s response to extreme climatic events such as drought and cold^26–29^.

Population-level differences in physiological response to drought could additionally have important implications for risk trade-offs in forest management. Reforestation with local seed sources is associated with risks because climate change is causing a mismatch of local plant populations to emerging climates. The rate of decoupling is expected to exceed the ability of long-lived forest trees to acclimate, migrate through dispersal or gene flow, or adapt through natural selection^30–32^. While trailing-edge populations are expected to accumulate extinction debts^32^, decreased forest health and productivity can be expected throughout a species' range due to locally adapted populations being genetically maladapted to new climate conditions^24^. Designed to overcome these issues, assisted migration is an adaptation strategy involving planting a percentage of pre-adapted seed sources from warmer, drier populations^25,33^. While this approach may help realign tree populations with their historic climatic optima, a careful assessment of the nature of adaptation is critical to effectively evaluate potential risks with longer-distance seed transfers^29,34^.

Recent work quantifying genetic differences in drought tolerance in terms of annual growth loss suggested northern boreal pine forests are most at-risk to drought under additional warming^27^. Meanwhile, central populations were moderately drought tolerant. However, explaining the underlying physiological mechanisms behind these responses is key for assessing vulnerability of forests to drought. This is required for vegetation modeling and accurate growth predictions as new climate assemblages appear and for assessing the efficacy of assisted migration.

An excellent tree species for studying the effects of climate change, climatic extremes and assisted gene flow is lodgepole pine (*Pinus contorta* Dougl. ex Loud.). Being one of the most common tree species in its native range of western North America, it has an extensive distribution covering ~1.3 million km^35^ in diverse climates. Due to its ecological and economic value, an extensive provenance trial was established in western Canada as a reciprocal transplant experiment in 1974^36^. Given the age of the trial, it can now be combined with tree-ring analyses to study genetic differences to annual events, including extremes such as drought and cold events.

Here, we test the physiological ability of different lodgepole pine populations to acclimate to warmer and drier conditions under climate change. We evaluate long-term growth, as well as annual losses in productivity following a severe spring drought that occurred in western North America in 2002. Growth responses were assessed by measurements of total tree height, annual height increments and tree-ring widths. The physiological mechanisms underlying the growth response to drought are assessed by comparing relative contributions of xylem adaptations and stomatal regulation. These are inferred by combining functional wood anatomical and dual-isotope analyses focusing on a 10-year window capturing the 2002 drought. Samples are collected from lodgepole pine trees grown in three common garden experiments in British Columbia’s southern interior. The 20 provenances investigated here are sourced from across a 4000 km range and grouped into four populations designed to represent major climate regions. By combining population genetic research with retrospective insights into growth, physiological and wood anatomical responses to drought, this study contributes an analysis of complex drought adaptations in a wide-ranging tree species under realistic field conditions. Northern trees produce more vulnerable hydraulic systems combined with lower stomatal responsiveness. This maladaptive response to drought means that western boreal forests may not cope well with droughts expected to increase in frequency with climate change.

## Results

### Differences in stable isotopes and xylem properties

**Fig. 1:**
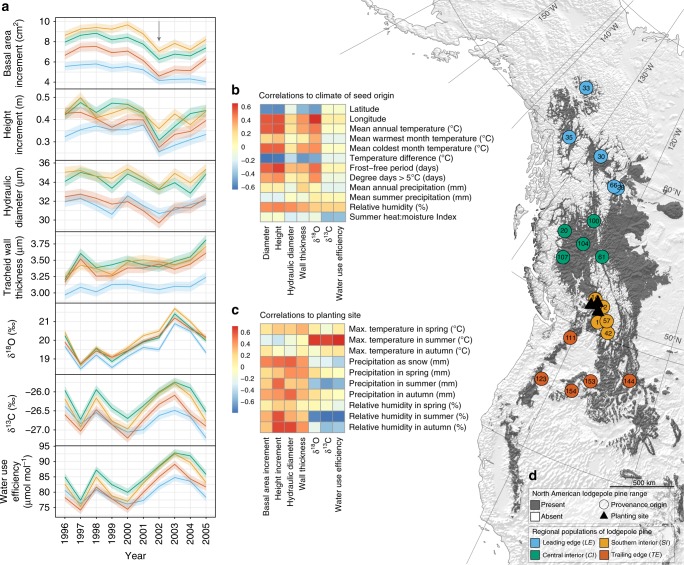
Variations in growth and physiology of lodgepole pine (*Pinus contorta*) in relation to climate and provenance. **a** Growth, functional wood anatomical traits, stable isotope values and δ^13^C-derived intrinsic water-use efficiency. Lines and standard errors (ribbons) are colored according to the regional population within the species distribution: blue represents the leading edge (northern) population; green represents the central interior population; yellow represents the southern interior population and orange represents the trailing-edge (far southern) population (*n* = 1170: 4 populations × 5 provenances × 1 tree × 3 sites × 2 blocks × 10 years, minus 3 trees). Grey arrow indicates drought year. **b** Correlations of growth and physiological traits to climate of seed origin. These were based on total height and diameter at age 32 and were correlated to long-term climates from 1961–1990 (*n* = 117). **c** Correlations of growth and physiological traits to annual climate variables at the planting sites. These were based on annual height and basal area increments related to weather on the planting sites over the 10 years (*n* = 600). **d** Lodgepole pine distribution (dark grey) in western North America and the experimental design. The map was produced by the authors with ArcInfo 10.1 using vector and raster data from www.naturalearthdata.com (Public Domain)

Physiological adaptations to drought varied greatly among the four regional populations of lodgepole pine. These pronounced differences in growth and physiological parameters were also maintained over the duration of the study (Fig. 1a). Seed sources from the central part of the lodgepole pine distribution showed consistently higher growth rates, hydraulic diameters of the xylem conduits as well as higher δ^13^C and δ^18^O values (Fig. 1a). In contrast, seed sources from northern areas of the lodgepole pine range showed consistently lower growth, hydraulic diameter of the xylem, and δ^13^C and δ^18^O values (Fig. 1a). Far southern provenances grew slower than central interior and southern interior populations (Fig. 1a). They also had moderate values in all physiological traits relative to central populations, except they had relatively thicker cell walls (Fig. 1a).

### Local adaptation and climate correlations

Responses of the four populations were associated with the climate conditions at the origin of the seed sources (Fig. 1b, Supplementary Tables 1, 3), indicative of local adaptations. Responses were also linked to interannual variation of climate at the planting sites (Fig. 1c, Supplementary Tables 2, 4), suggesting plastic responses. Growth, tracheid wall thickness and δ^18^O showed stronger latitudinal clines of adaptation linked to temperature and growing season conditions (Fig. 1b). In contrast, the δ^13^C and intrinsic water-use efficiency (iWUE) values were found to be more linked to site climate conditions than could be explained by climate of seed origin (Figs. 1b, 1c, Supplementary Tables 3, 4). We find a positive correlation between δ^18^O and δ^13^C among all populations studied here but note that the northern population has less variation in δ^13^C (Supplementary Figure 1). The positive relationships generally indicate that δ^13^C is governed more by stomatal conductance than photosynthesis given the importance of relative humidity in our study (Fig. 1c).

### Total growth and drought tolerance

**Fig. 2:**
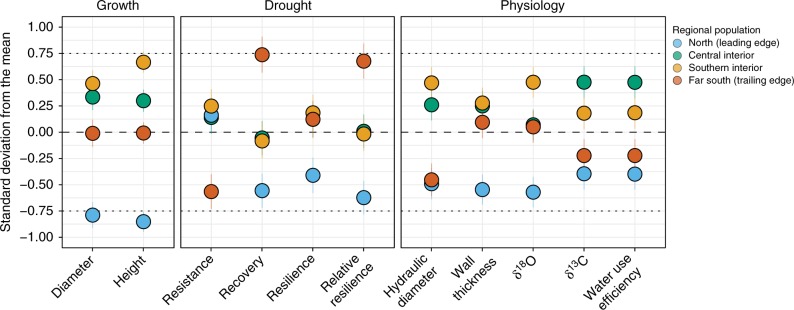
Genetic adaptations revealed from growth, drought tolerance and physiology. Each dot represents the average response in units of standard deviation to show relative rankings among the four regional populations (*n* = 1170: 4 populations × 5 provenances × 1 tree × 3 sites × 2 blocks × 10 years, minus 3 trees). Regional populations are coloured as such: blue represents the leading edge (northern) population; green represents the central interior population; yellow represents the southern interior population; and orange represents the trailing-edge (far southern) population. Response is tested across three planting sites in British Columbia’s southern interior. Therefore a positive climate transfer distance exists for the northern population (tests climate warming) while a negative climate transfer distance exists for the southern population (tests assisted migration scenarios). Bars are standard errors of the mean

Total height and diameter growth, representing average metrics of overall success, were compared to drought-tolerance indicators and 10-year averages of physiological parameters in Fig. 2. Higher productivity and moderate drought tolerance of central and southern interior populations were linked to higher δ^13^C and δ^18^O values, larger hydraulic diameter, and thicker cell walls (Fig. 2). Values for these traits were almost always significantly different from the northern, leading-edge population (Fig. 2, Table 1, Supplementary Tables 6-9). The far southern, trailing-edge population showed the highest drought tolerance of all four populations, as measured by recovery and relatively resilience following the 2002 drought (Fig. 2). Relative to the most productive central and southern interior populations, they also showed moderate productivity linked with significantly lower tracheid hydraulic diameter (Fig. 2, Table 1 Supplementary Tables 6, 9). The trailing-edge population had lower average iWUE as well as lower δ^13^C and δ^18^O values compared to central populations (Fig. 2, Table 1, Supplementary Table 8). In contrast, the northern, leading-edge population showed the lowest values for average growth, δ^13^C and δ^18^O, and for xylem hydraulic properties—notably, significantly thinner tracheid walls on average (Fig. 2, Table 1, Supplementary Table 9).

**Table 1:** Multiple comparisons of growth, drought indicators and physiological traits in interior lodgepole pine populations

Test	BAI	HI	Resis.	Recov.	Resil.	Rel. Resil.	Hydr. Diam.	Wall. Thick.	δ^18^O	δ^13^C	iWUE
LE–CI =0	< **0.001**	< **0.001**	> 0.999	0.130	0.145	**0.033**	**0.011**	**0.002**	**0.025**	**0.001**	**0.001**
SI–CI = 0	0.481	0.999	0.968	0.999	0.995	> 0.999	0.927	0.985	0.570	0.674	0.678
TE–CI = 0	0.108	0.104	**0.016**	**0.004**	> 0.999	**0.025**	**0.023**	0.811	0.999	**0.018**	**0.018**
SI–LE = 0	**<** **0.001**	**<** **0.001**	0.983	0.176	0.087	**0.048**	< **0.001**	**0.005**	< **0.001**	**0.038**	**0.036**
TE–LE = 0	0.144	**0.013**	**0.012**	< **0.001**	0.156	< **0.001**	0.999	**0.033**	**0.035**	0.882	0.876
TE–SI = 0	**0.001**	0.082	**0.004**	**0.003**	0.995	**0.019**	**0.002**	0.948	0.522	0.241	0.239

Multiple comparisons among tree populations representing the full north-south range of interior lodgepole pine (*Pinus contorta* ssp. *latifolia*). Populations are represented by five provenances grouped into four climatic regions, LE, CI, SI and TE: LE stands for the Leading Edge, i.e. the northern population occupying the area expected to be the leading edge of tree species migrations under climate warming; CI is Central Interior population, located in the central areas of the lodgepole pine range; SI is the Southern Interior population covering the southern range of the central areas of the lodgepole pine range; TE stands for the Trailing Edge, which represents seed sources from the far south of the lodgepole pine range, which is expected to see increased forest maladaptation under climate warming

*BAI* is basal area increment, *HI* is height increment, *Resis.* is drought resistance, *Recov.* is drought recovery, *Resil.* is drought resilience, *Rel. Resil.* is drought relative resilience, *Hydr. Diam. *is mean hydraulic diameter, *Wall. Thick.* is mean tracheid wall thickness, and *iWUE* is intrinsic water-use efficiency derived from tree-ring δ^13^C. Significance (*α* = 0.05) is indicated in bold and *p*-values were adjusted with the Benjamini & Hochberg false discovery rate method

### Physiological plasticity under drought

**Fig. 3:**
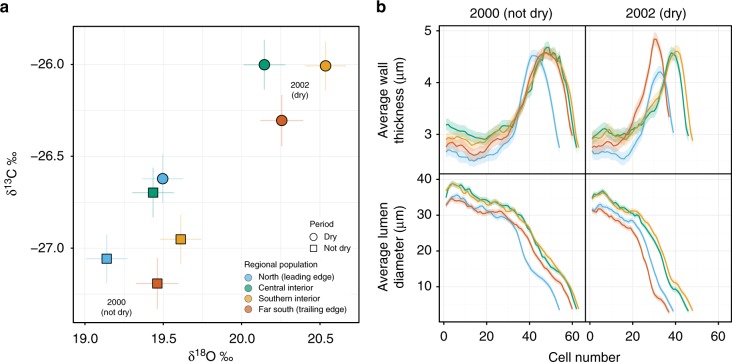
Plasticity among populations of lodgepole pine. **a** Response of stable isotopes in two years of different moisture levels. **b** Average tracheid wall thickness and lumen diameter as a function of cell number under different moisture conditions in the year 2000 (not dry) and 2002 (dry). Each of the regional populations are colored according to Fig. 1. Based on *n* = 1170 (4 populations × 5 provenances × 1 tree × 3 sites × 2 blocks × 10 years, minus 3 trees). Error bars (**a**) and ribbons (**b**) are standard errors of the mean

The plasticity of responses under contrasting climate conditions also varied among the four lodgepole pine populations studied here (Fig. 3). Relative to leading and trailing-edge populations, dual-isotope signatures of central interior and southern interior populations indicated a high degree of plasticity. These populations showed the greatest difference in values between dry and non-dry years (Fig. 3a). Under drought, the ratio of carbon-13 to carbon-12 increased while oxygen-18 became enriched relative to oxygen-16. Under drier conditions, however, these populations did not modify their xylem structure to the same degree as the trailing-edge (far southern) population (Fig. 3b). The far southern, trailing-edge population showed a moderate range in stable isotope values under contrasting conditions, but showed the most flexibility in adjusting their hydraulic properties (Fig. 3a, b). Under drought, far southern seed sources produced fewer tracheids, but those tracheids had smaller lumen diameters and thicker cell walls (Fig. 3b). In contrast, the northern population showed the lowest ability to modulate its response in terms of isotopic signatures or xylem parameters (Fig. 3a, b). Their cell walls also became thinner under drought (Fig. 3b).

## Discussion

Diverse physiological mechanisms underlie the differences observed in drought tolerances of divergent lodgepole pine populations. Wood anatomical responses and isotopic signatures revealed different relative contributions of xylem and stomatal control, suggesting varied strategies to cope with drought. The higher productivity of central populations can be linked to larger tracheid lumens, which form an efficient hydraulic pathway to the photosynthesizing crown. This facilitates growth when moisture is available, giving the trees a competitive advantage in photosynthesis and growth potential under optimal conditions^37^. At the same time, cavitation risk appears to be reduced through stomatal closure under drought, inferred through stable isotope signatures (Figs. 1–3). Central populations showed the greatest differences in dual-isotope signatures in response to changing climatic conditions but did not modify their xylem structure to the same degree as the trailing-edge (far southern) population (Fig. 3). The genetic history of the provenances may help explain the higher iWUE exhibited by the central interior group. This population shows introgression between the coastal sub-species (*P*. *contorta* ssp. *contorta*)^38^. For lodgepole pine, higher moisture at the seed origin has been linked to higher iWUE and growth potential^39,40^.

The drought-avoidant behavior exhibited by central and southern interior populations could explain their moderate resilience to drought^27^. This may provide the impression that forests in central and southern interior areas are not as urgently in need of assisted migration interventions relative to northern areas. However, their physiological plasticity may be limited, and the mechanisms may not continue to be successful under prolonged drought conditions because larger tracheids are more prone to cavitation. In a European pine species, for example, individuals that had produced larger tracheids after drought showed symptoms of dieback^41^. A common assumption is that stomatal sensitivity to drier conditions may be associated with increased risk of carbon reserves being depleted under extended drought^14^. A recent experiment on saplings, however, found that drought-induced mortality in an isohydric tree species was not linked to carbon starvation over a period of 3 years^42^. Nevertheless, carbon starvation could eventually lead to mortality, but more likely weakens the tree, making it susceptible to pest and pathogens as other mortality agents^43^. Therefore, decline of central and southern interior forests in the future remains a risk depending on the rate and magnitude of warming and drying, as well as interactions with other biotic and abiotic factors. Judicious northward seed transfer to central interior and south–central regions of the species range may therefore be warranted.

The drought-tolerant behaviour exhibited by trees from the southern, trailing-edge population was linked to different physiological behaviours compared to central populations. Far southern provenances showed some ability to adjust their hydraulic diameter, xylem cell lumen diameter and cell wall thickness in response to drier conditions. Stable isotope signatures suggest that while their stomata do respond to drought (Fig. 3a), they also, on average, leave their stomata open longer than central and southern interior populations (Fig. 2). Assuming respiration rates are not disproportionately greater^44^, this behavior may be better suited to cope with prolonged drought. By continuing to assimilate carbon, carbon starvation can be avoided, while cavitation risk is reduced through adjustments of the xylem. The investment in such safety mechanisms further explains the classic growth versus drought tolerance trade-off exhibited by the far southern provenances^27^. Planting a proportion of such southern seed sources in the south–central and central interior areas of the species distribution could therefore increase drought tolerance by introducing a different drought behaviour to those forests. While complementary physiological strategies could increase the adaptive portfolio of southern interior forests to drought, shorter transfer distances are recommended since maladaptation to cold could otherwise limit forest productivity^29^.

Most importantly, the northern population appears to be unsuitably adapted to cope with drought. The dual-isotope signatures suggest that they do not modify stomatal behaviour while, at the same time, their xylem cell walls become thinner under drought—a maladaptive response due to higher cavitation risk^45^. Under drought, the combination of sustained stomatal conductance and low cell wall thickness unsuited to compensate for increasing xylem tension would likely cause the northern population to be more cavitation prone. This is consistent with their low drought tolerance; these trees did not regain pre-drought performance. Interestingly, these data also show no evidence of an expected dual adaptation to cold and drought tolerance^46^, which may rely on similar mechanical properties. The smaller lumen diameters could be an avoidance strategy against embolisms caused by drought or freeze–thaw events. However, the relatively thin cell walls of northern populations observed in this study would have an opposite effect: low drought resilience for northern populations of lodgepole pine.

Trade-offs affecting phenology, wood formation and growth response to drought may also exist for each of the four populations. Trees that flush and begin the process of xylogenesis early in the growing season may risk late spring frosts, but, at the same time, may have a competitive advantage by capturing early season moisture. As annual growth dynamics vary among populations, the isotope patterns may also vary accordingly. However, the drought in 2002 occurred early in the growing season. Therefore, all provenances can be assumed to have been affected during wood formation, which would be reflected in isotopic signatures as well. Although all trees studied here were grown in common environments, genotypes that begin growing earlier may acquire slightly different oxygen isotope signatures from melting snow. When northern provenances are grown in warmer planting environments, they appear to meet their heat-sum requirements early, which can lead to cold damage in spring under climate change^29^. However, by comparing the responses of the four populations in a drought and non-drought year (Fig. 3), it is evident that the northern population shows the most limited plasticity in response, which appears to be less likely due to phenological differences.

Finding such differences among tree populations further highlights the value of combining genetic field experiments with tree-ring approaches to evaluate intraspecific variation of physiological responses to drought. Assessing annual responses of tree populations grown in multi-decade provenance trials provides an informative approach to determining adaptive capacity to extreme events that are expected to become more prevalent in the future. The reconstruction of annual growth, height increment, wood anatomical and ecophysiological behavior in combination with genetic provenance testing provides insight into the nature and geographic distribution of drought adaptation. This method could be applied to other long-term reciprocal transplant experiments that have been established around the world to infer limits to adaptive capacity within forest species.

Forest health issues, productivity losses and carbon-cycle feedbacks are unlikely to be uniform across tree species distributions due to physiological adaptations at the population level. Counterintuitively, our study shows that northern pine forests will experience the consequences of forest maladaptation first, due to an inability to cope with drought. This suggests that the potential benefit of increased growth due to warming may be counterbalanced by physiological maladaptation to drought, contributing to negative predictions for the western boreal. Intervention in northern forests therefore appears to be more urgent than elsewhere in the species range. In northern forests, therefore, introducing pre-adapted alleles to the leading edge may be a low-risk solution targeting forest maladaptation under climate change.

## Methods

### Experimental design

All trees used in this study were grown in the Illingworth provenance trial, a genetic field experiment established in 1974 for lodgepole pine^36^. Many planting sites incurred high mortality in 2006 due to the mountain pine beetle epidemic. We were therefore permitted to fell dead trees within this valuable multi-decade experiment in 2013 and 2014, which allowed to accurately measure growth parameters and collect stem disks. The family structure in the Illingworth trial was not maintained; provenances (seed sources) represent stand samples as opposed to single progenies. Although several sub-species exist within the lodgepole pine range, we sampled provenances originating from the drier interior region of the range represented by the economically important timber sub-species, *P. contorta* ssp. *latifolia*. From a genetics history perspective, one provenance from the far south-west of the range (provenance 123) may show introgression with *P.contorta* ssp. *murrayana*, while some central interior provenances may show introgression with the coastal variety, *P. contorta* ssp. *contorta*^38^.

To facilitate analyses, we grouped provenances to account for different climatic regions as well as known patterns of genetic differentiation in adaptive traits. The experimental design was not based on genotyping, but followed clinal variations in lodgepole pine growth and adaptive traits, which are steep in elevation but gentle across latitude^40,47^. Provenances were thus grouped into four geographic regions (populations): (1) the boreal North representing the cold leading edge (LE) of the species range; (2) the cool central interior (CI) area of the species range; (3) warm, lower-elevation southern interior (SI) areas of the species range; and (4) the trailing-edge (TE) group represents the most southern samples of the provenance trial in the United States, with the warmest and driest climatic conditions. Including the leading and trailing-edge populations was necessary to inform our understanding of how these important populations will respond to climate change and to evaluate assisted migration scenarios from a drought perspective.

Each of the four regional populations is represented by five provenances, grown on three planting sites in British Columbia’s southern interior and replicated on two blocks per site in a randomized block design (Fig. 1d, Supplementary Figure 2, Supplementary Tables 1 and 2). Growth and drought-tolerance data from a previous study relied on 4 trees per provenance per block^27^. Due to the high financial and labour costs involved in stable isotope and functional wood anatomical analyses, however, this work relies on a sub-sample of 117 trees. This sub-sample is represented by the tree of median height per provenance (20) per block (2) per site (3). Three samples were not available.

### Field and tree-ring measurements

After felling and de-limbing each tree, we used an Eslon tape to measure height and annual height increment, indicated by the distance between branch whorls. We then cut stem disks for further growth and physiological analyses. In the field, we confirmed the accuracy of annual height increment counts by comparing them to the number of tree rings from stem disks at a given height. Although we were limited to sampling trees killed by mountain pine beetle, future work characterizing drought adaptations in tree populations could quantify leaf-to-sapwood area ratios in living trees in provenance trials.

After all data was obtained, the accuracy of height increments was again verified against the same tree’s tree-ring widths. All tree-ring, wood anatomical and isotopic analyses presented here are based on stem disks taken from diameter at breast height (1.3 m). After sanding and scanning these disks, we measured annual radial growth on four radii per stem disk using Windendro (version 2016) to a precision of 0.01 mm and derived basal area increment (BAI). Stem disks were then cut into two adjacent sections of ~1 cm along a radius to the pith: one each for functional wood anatomical and stable isotope analyses.

### Drought indicators and period of study

A study period of 10 years from 1996 to 2005 was chosen as it captured a spring and summer drought event occurring in 2002. Compared to the 1961–1990 climate normal, this drought represented a drop of one standard deviation in mean annual precipitation, while standard deviations from mean precipitation in June, July, August, and September were −0.61, −0.62, −0.90, and −0.75, respectively. Designation of these years as pre-drought, drought and post-drought are important for the super-epoch analysis involved in calculating the four drought indicators, as coined by Lloret et al.^48^: Resistance, refers to the drop in growth in the drought year relative to the three preceding years; recovery is the speed with which growth resumes; resilience is the ability to return to pre-drought growth; and relative resilience is the ability to return to pre-drought growth relative to the severity of the growth drop during the drought year. These variables were defined with regard to BAI to be consistent with an earlier study^27^, where 1999–2001 are considered pre-drought years, 2002 is the drought year and 2003–2005 are post-drought years. Although 2003 was also considered warm, it was not quite as dry in the early part of the growing season when growth is critical. Since BAI had already begun to recover in 2003, by definition, it was considered a post-drought year.

### Functional wood anatomy

After applying a corn-starch solution to the wood sections^49^, we cut micro sections of 10–20 µm thickness using a GSL-1 microtome^50^. The samples were washed, steamed^51^, rinsed and bleached^52^. The samples were then soaked in a stain made of equal portions of Safranin and Astrablue for 5 min, rinsed with distilled water, and washed with increasing concentrations of ethanol^52^. After applying Canada balsam, the samples were covered with glass cover slips and placed in an oven at 60 °C for 8 h to solidify. Micrographs were taken at ×200 magnification at a resolution of 5 megapixels and 12-bit colour depth using a Nikon Eclipse Ni-E upright microscope and automatically stitched together with the NIS-Elements software, version 4.20.1. Images were further cleaned as necessary in Photoshop (Adobe CS4) or WinCell, version 2016.

For each tree ring, four cell rows were measured in WinCell to derive average cell lumen diameter and cell wall thickness. We subsequently conducted wood anatomical analyses which provide data on and insight into physiological processes. These include the hydraulic diameter for each ring (equation 1), a measure of the xylem’s potential as a water pipeline to the photosynthesizing crown^53^:

1 $$\sum {\mathrm{d}}^{\mathrm{5}}/\sum {\mathrm{d}}^{\mathrm{4}}$$

Relative comparisons among populations are possible since differences in xylem anatomy due to shade stress are largely accounted for by using the provenance trial setting, where trees were grown in a common age structure.

### Pilot: confirm methodology for stable isotope analyses

Regulation of water loss through reduced stomatal aperture also affects the stable isotope ratios (i.e. δ^13^C or δ^18^O) in photosynthate used to form secondary growth^54–57^. Water-stressed conifers have a reduced ability to discriminate against the disfavored heavy carbon-13 isotope relative to the more abundant light carbon-12 isotope, and leaf water becomes increasingly enriched in the heavy oxygen-18 isotope. A positive correlation between δ^13^C or δ^18^O points to stomatal closure rather than changing rates of photosynthesis^55,58^. With an awareness of other complexities affecting isotope composition of tree rings^55–57^, a dual-isotope approach can be used to retrospectively infer stomatal responsiveness to drought.

We conducted a pilot study to confirm the most suitable methodology for annually-resolved isotopic analysis on our lodgepole pine samples. It has long been recognized that wood components vary isotopically^59–61^. The ratio of lignin to cellulose in a tree ring may therefore alter the stable isotope ratios^59^. Cellulose is often used for stable isotope analysis due to its stability and immobility^60,62,63^. However, cellulose extraction is time-consuming, so whole-wood is preferred when there is a consistent offset. For lodgepole pine, Guy and Holowachuk^39^ previously reported a strong relationship between carbon isotope signatures in cellulose and whole sapwood. Here, however, it was important to test the reliability of this signal over time in tree rings, the key component in our study, from trees that had been dead-standing for 5–8 years for both oxygen, as well as carbon isotope ratios. We also wanted to exclude the influence of blue-stain fungus affecting many of our lodgepole pine samples on the isotope ratios, as reported for other conifers^64^. This pilot additionally compared latewood to the entire ring to test if earlywood cells were formed with stored photosynthate, possibly leading to different climatic correlations^65,66^.

The pilot study was based on tree rings covering 7 years (1999–2006, inclusive), representing four trees from one provenance grown on one planting site block. Tree rings were separated with a scalpel under a stereo microscope (Leica Wild M3B, Wetzlar, Germany). Whole-wood samples were homogenized with an ultra-centrifugal mill (ZM 2000, Retsch GmbH, Germany) and placed in Teflon sample bags. To remove possible resins and extractives, the bags were washed continuously with 96%-grade ethanol for 24 h in a Soxhlet apparatus. For cellulose samples, tree rings were placed into Teflon bags and hollocellulose was extracted following a modified Jayme-Wise method^62^. This procedure involved placing the samples in a bath of 5% sodium hydroxide (NaOH) for 2 h at 60 °C, then thoroughly rinsing the samples. Samples were placed in a bath of 7% sodium chlorite (NaClO_2_) and then glacial acetic acid for 30 h at 60 °C, the average time for samples weighing 10 mg.

Supplementary Figure 3 shows stable isotope values over time derived from cellulose, whole-wood, latewood and entire-ring samples. Cellulose and whole-wood year-to-year variations of pilot samples were highly correlated (*r* = 0.92 for δ^13^C; *r* = 0.85 for δ^18^O) but were significantly different (*p* < 0.001 for both δ^13^C and δ^18^O, two-sided paired *t*-test). Isotope ratios for latewood and the entire ring were also highly correlated for δ^13^C (*r* = 0.88) and significantly different (*p* = 0.009, two-sided paired *t*-test). The correlations between latewood and the entire ring were weaker for δ^18^O (*r* = 0.39), but these groups were not significantly different from each other for δ^18^O (*p* = 0.701, two-sided paired *t*-test). Latewood, however, showed higher variability. This could be due to inconsistencies introduced when separating the latewood from the earlywood with a scalpel; splitting was assessed visually and was therefore subjective. Using the entire ring was therefore considered more suitable, especially as it represents a time-integrated response and had stronger correlations with climate. This pilot study confirmed that using the entire tree ring and extracted whole-wood were most suitable for these stable isotope ratio analyses.

### Tree-ring sample preparation for stable isotope analyses

For the full design, we removed extractives from the wood sections by submerging them in ethanol in a Soxhlet apparatus for 24 h. We then separated tree rings with a scalpel under a stereo-microscope (Leica Wild M3B, Wetzlar, Germany) and homogenized the samples with an ultra-centrifugal mill (ZM 2000, Retsch GmbH, Germany). This homogenization technique is appropriate for whole wood and poses no risk of isotopic contamination from plastic sample tubes, as compared to some ball mill homogenization methods^67^. The homogenized samples were weighed into tin (carbon) and silver (oxygen) capsules for elemental analyses and mass spectrometry. Carbon samples were flash combusted at 1800 °C while oxygen samples were pyrolised at 1450 °C before being fed into the Isotope Ratio Mass Spectrometry interface. The resulting isotope ratios are reported in standard δ notation, which represents deviations from the reference material (Vienna Pee Dee Belemnite for Carbon; Vienna Standard Mean Ocean Water for Oxygen) according to eq. 2:

2 $${\mathrm{R}} = \left( {{\mathrm{R}}_{{\mathrm{sample}}}{\mathrm{/R}}_{{\mathrm{reference}}}-{\mathrm{ 1}}} \right){\mathrm{1000}}$$

Where *R* is the ratio of the heavier to lighter isotope. The level of precision is ± 0.02‰ for carbon and ± 0.4‰ for oxygen. Assuming no differences in rooting structure among populations, relative differences among populations’ δ^18^O signatures point more to transpiration than water source due to the common garden approach and, hence, relatively homogenous soil water conditions.

### Atmospheric carbon data and iWUE

Although the isotopic signatures may change slightly due to post-assimilation fractionation^56,57^ and that stored carbohydrate reserves can be remobilized, stable carbon isotope ratios can still provide information on stomatal behaviour and are widely used. We used tree-ring δ^13^C to estimate atmospheric and intercellular carbon concentrations (*c*_*a*_ and *c*_*i*_, respectively), which were used to calculate iWUE based on the following empirically-derived eq. 3 and 4 ^61,68^:

3 $$c_i/c_a = \left( {{\mathrm{\delta }}^{{\mathrm{13}}}{\mathrm{C}}_{{\mathrm{plant}}}-{\mathrm{\delta }}^{{\mathrm{13}}}{\mathrm{C}}_{{\mathrm{air}}} + a} \right)/\left( b - a \right)$$

4 $$_{\mathrm{i}}{\mathrm{WUE}} = A/g_s = c_a\left[ {{\mathrm{1}} - \left( {c_i/c_a} \right)} \right]\left( {{\mathrm{0}}{\mathrm{.625}}} \right)$$

Where *a* represents a fractionation of −4.4‰ due to kinetic diffusion discrimination at the stomata, *b* is the fractionation at the site of carboxylation (−27‰). The rate of carbon assimilation is denoted by *A*, and *g*_*s*_ represents stomatal conductance. Annual mean estimates for *c*_*a*_ were obtained from the United States Department of Commerce’s National Oceanic and Atmospheric Administration Earth System Research Laboratory observations from Mauna Loa^69^ (ftp://aftp.cmdl.noaa.gov/products/trends/co2/co2_annmean_mlo.txt). Annual estimates for δ^13^C_air_ were downloaded from the flask analysis data for Mauna Loa, available from the Carbon Dioxide Information Analysis Center^70^ (CDIAC; http://cdiac.ornl.gov/ftp/trends/co2/iso-sio/).

### Climate data

All climate data were derived from the ClimateWNA (version 5.4) software interface^71^. We extracted annual climate variables for planting sites. For the provenance climates (climate of seed origin), we aim to approximate the climates to which the tree populations are adapted. We derived climate variables for the 1961–1990 long-term average. This climate normal is the earliest period with a high density of climate data and precedes the most recent anthropomorphic warming trend.

### Statistical analyses

All statistical analyses were conducted in the R statistical programming environment^72^, and graphing was implemented with various functions from the ggplot2 package^73^. Correlations to climate at the planting site were conducted at the provenance level and were calculated with the rcorr function from the Hmisc package^74^ to obtain *p*-values. Due to the slightly imbalanced design, provenance values for the correlations relied on least square means calculated using lsmeansLT and lmerTest^75^, where provenance was specified as the fixed effect, and block and a unique tree identity were specified as random effects. For testing differences among the four regional populations, the fixed effect in each model was region. Model selection was verified by confirming lower values for Akaike’s Information Criteria.

For models based on functional wood anatomy, the distance from the apex was included as a random effect because it affects conduit size^76^. It is necessary to include distance to the top of the tree since conduit size is partially determined by growth patterns over time. As the tree grows higher, the new growth at the base of the tree requires larger conduits^76^. This is therefore reflected in functional wood anatomical data in a gradient towards the pith in a stem disk or increment core^76^. Correlations between growth and annual climate conditions at the site were based on height increments and BAIs. Correlations of the average climate of seed origin (the long-term mean from 1961–1990) to all growth, drought, anatomical and isotopic traits are designed to show adaptation to climate. For these correlations, we used total height and diameter at breast height after 32 years of growth in real-world conditions (year 2005). All correlations were also adjusted using the Benjamini & Hochberg false discovery rate method^77^ for reporting significance in tables (Table 1, Supplementary Tables 6-9). Heatmaps were produced using the pheatmap command in the pheatmap package^78^. Post-hoc tests for regional population fixed effects were completed with the glht function from the multcomp package^79^ and the *p*-values were adjusted using the Benjamini & Hochberg false discovery rate method as well^77^.

## Electronic supplementary material

Supplementary Information

Reporting Summary

## Data Availability

All relevant data is available from the authors.
