# Publisher Correction: Northern forest tree populations are physiologically maladapted to drought

**DOI:** 10.1038/s41467-020-15177-0

**Published:** 2020-03-09

**Authors:** Miriam Isaac-Renton, David Montwé, Andreas Hamann, Heinrich Spiecker, Paolo Cherubini, Kerstin Treydte

**Affiliations:** 1 Department of Renewable Resources, University of Alberta, Edmonton, AB T6G 2H1 Canada; 2 Chair of Forest Growth and Dendroecology, Albert-Ludwigs-Universität-Freiburg, Tennenbacherstrasse 4, 79106 Freiburg, Germany; 3 Swiss Federal Institute for Forest, Snow and Landscape Research WSL, Zürcherstrasse 111, CH-8903 Birmensdorf, Switzerland

**Keywords:** Population genetics, Drought, Climate-change impacts, Boreal ecology, Ecophysiology

Correction to: *Nature Communications* 10.1038/s41467-018-07701-0, published online 10 December 2018.

The original version of this Article contained an error in Fig. 1, in which the labels for Fig. 1b and Fig. 1c were incorrectly swapped. This has been corrected in the PDF and HTML versions of the Article.

