# Supplementary material for: Northern forest tree populations are physiologically maladapted to drought
Source: Nat Commun. 2018 Dec 10;9:5254. doi: 10.1038/s41467-018-07701-0 (PMC6288165; doi:10.1038/s41467-018-07701-0)
Supplement: Supplementary file 1 — Supplementary Information [file 41467_2018_7701_MOESM1_ESM.pdf]

Supplementary Information

Northern forest tree populations are physiologically maladapted to drought

Isaac-Renton *et al.*

Supplementary Tables

Supplementary Table 1. Geography and average climate variables for all provenances

Region	Prov	Name	Lat	Long	Elev	MAR	MAT	MWMT	MCMT	TD	MAP	MSP	PAS	RH
North (Leading Edge)	33	Ethel L.	63.30	-136.47	876	9.8	-4.2	14	-24.7	38.7	442	260	198	57
North (Leading Edge)	30	Lower Post	59.98	-128.55	640	9.7	-2.7	14.5	-23.5	38	448	235	204	56
North (Leading Edge)	35	Atlin	59.80	-133.78	789	9.8	-0.6	12.5	-15.5	28	374	183	181	63
North (Leading Edge)	28	Tetsa R.	58.67	-124.17	762	10.5	-1.2	13.7	-18.2	31.9	621	419	195	58
North (Leading Edge)	66	Stone Mt.	58.65	-124.77	1173	10.1	-1.3	11.3	-14.4	25.7	688	475	240	61
Central Interior (Central to Distribution)	100	Nina Cr.	55.80	-124.82	762	10.9	0.9	13.5	-14	27.5	529	250	234	61
Central Interior (Central to Distribution)	20	Collins L.	54.13	-127.23	937	11.1	2	12.5	-9.4	21.9	595	219	294	64
Central Interior (Central to Distribution)	104	Nechako R.	54.02	-124.53	732	11.1	2.6	14.4	-11.4	25.7	520	238	211	58
Central Interior (Central to Distribution)	107	Tweedsmuir	52.50	-125.80	1311	11.3	1.2	10.9	-8.8	19.8	1043	257	628	60
Southern Interior (Central to Distribution)	14	Wentworth Cr.	50.97	-120.33	1059	12.3	4.2	14.8	-7.6	22.4	476	219	163	62
Southern Interior (Central to Distribution)	72	Larch Hills	50.70	-119.18	777	11.2	5.8	17.1	-5.9	23	648	244	211	65
Southern Interior (Central to Distribution)	57	Inonoaklin	49.90	-118.20	579	10.7	6.8	17.9	-4.4	22.3	685	264	162	64
Southern Interior (Central to Distribution)	1	Trapping Cr.	49.58	-119.02	1006	11.1	4.5	15.7	-7.1	22.8	550	227	200	59
Southern Interior (Central to Distribution)	42	Champion L.	49.18	-117.58	998	12.7	6.2	17.6	-5.2	22.8	835	269	261	67
Far South (Trailing Edge)	111	Stevens Pass	47.78	-120.93	762	16.5	6.5	17.2	-3.5	20.7	1711	255	536	61
Far South (Trailing Edge)	144	Missoula (a)	46.67	-113.67	1524	12.7	4.7	16.8	-6.3	23.1	568	237	199	56
Far South (Trailing Edge)	153	Enterprise	45.63	-117.27	1311	14.5	6.7	17.7	-3.4	21.1	562	205	123	56
Far South (Trailing Edge)	154	Prairie City	44.53	-118.57	1494	15.9	6	17.1	-4.1	21.3	639	171	206	56
Far South (Trailing Edge)	123	Black Butte	44.38	-121.67	1006	14.3	7.3	16.6	-0.7	17.3	609	102	99	51

Climate variables represent the long-term average from 1961 to 1990. Region refers to the assigned population based on geographical origin. Prov refers to the provenance corresponding to the Illingworth provenance trial's numbering system: Latitude (Lat) and longitude (Long) are given decimal degrees; elevation (Elev) is given in meters above sea level, and MAR represents Mean Annual Solar Radiation (MJ m^{-2} d^{-1}). Climate variables include: Mean Annual Temperature (MAT, °C), Mean Warmest Month Temperature (MWMT, °C), Mean Coldest Month Temperature (MCMT, °C), Temperature Difference (TD, °C), Mean Annual Precipitation (MAP, mm), Mean Summer Precipitation (MSP, mm), Precipitation as Snow (PAS, mm) and Relative Humidity (RH, %).

Supplementary Table 2. Geography and average climate variables of the three planting sites

Site	Lat	Long	Elev	MAR	MAT	MWMT	MCMT	TD	MAP	MSP	PAS	RH
Chuwels Lake (CHUW)	50.58	-120.62	1430	12.7	2.9	13.5	-7.2	20.7	445	214	164	63
Community Lake (COMM)	50.92	-120.07	1370	12.6	3.3	14.1	-7.0	21.1	664	280	262	67
Equises Creek (EQUI)	50.37	-119.60	1370	12.3	3.5	14.1	-7.1	21.2	743	270	308	66

Climate variables represent the long-term average from 1981 to 2010, encompassing the study period. The three planting sites are within the Illingworth lodgepole pine provenance trial. Latitude (Lat) and longitude (Long) are given decimal degrees; elevation (Elev) is given in meters above sea level, and MAR represents Mean Annual Solar Radiation (MJ m^{-2} d^{-1}). Climate variables include: Mean Annual Temperature (MAT, °C), Mean Warmest Month Temperature (MWMT, °C), Mean Coldest Month Temperature (MCMT, °C), Temperature Difference (TD, °C), Mean Annual Precipitation (MAP, mm), Mean Summer Precipitation (MSP, mm), Precipitation as Snow (PAS, mm) and Relative Humidity (RH, %).

Supplementary Table 3. Pearson's correlation coefficients of provenance growth, drought tolerance and physiology to climate of seed origin

	Growth		Drought Tolerance				Physiology				
	Diameter (cm)	Height (m)	Resistance	Recovery	Resilience	Relative Resilience	Hydraulic Diameter (µm)	Tracheid Wall Thickness (µm)	$\delta^{18}O$ (‰)	$\delta^{13}C$ (‰)	iWUE ($\mu mol\ mol^{-1}$)
Latitude	**-0.53**	**-0.53**	0.39	**-0.76**	-0.44	**-0.75**	-0.10	-0.40	-0.48	0.02	0.02
Longitude	**0.65**	**0.66**	-0.24	0.43	0.21	0.41	0.30	0.49	**0.74**	0.18	0.18
Mean Annual Temperature (°C)	**0.63**	**0.65**	-0.26	**0.70**	0.49	**0.71**	0.25	0.45	**0.56**	0.04	0.04
Mean Coldest Month Temperature (°C)	0.30	0.38	-0.15	0.43	0.25	0.37	0.03	0.16	0.42	-0.10	-0.10
Mean Warmest Month Temperature (°C)	**0.61**	**0.62**	-0.34	**0.76**	0.50	**0.78**	0.22	0.47	0.51	0.05	0.05
Temperature Difference (°C)	**-0.61**	**-0.59**	0.34	**-0.74**	-0.50	**-0.78**	-0.25	-0.49	-0.44	-0.10	-0.10
Frost-free period (No. of days)	**0.61**	**0.69**	-0.05	0.26	0.17	0.22	0.38	0.41	**0.59**	0.13	0.13
Degree days > 5 °C (No. of days)	0.45	**0.51**	-0.13	**0.50**	0.36	0.47	0.15	0.26	0.51	-0.04	-0.04
Mean Annual Precipitation (mm)	0.16	0.17	-0.31	0.35	0.07	0.31	-0.08	0.32	0.11	-0.12	-0.12
Mean Summer Precipitation (mm)	-0.13	-0.14	0.09	**-0.57**	-0.51	**-0.59**	0.04	-0.04	0.18	0.12	0.12
Relative Humidity (%)	**0.50**	**0.53**	0.04	-0.23	-0.19	-0.22	0.51	0.43	0.32	0.32	0.32
Summer Heat:Moisture Index	0.00	0.02	-0.05	**0.63**	**0.63**	**0.69**	-0.18	-0.12	-0.06	-0.38	-0.38

Climate of seed origin represents the long-term average from 1961 to 1990. Growth variables include: total diameter in centimeters in year 2005, when trees had grown in real world conditions for 31 years (Diameter) and height in year 2005 in meters (Height). Drought tolerance variables include: drought resistance, as measured by the loss of growth in a drought event (Resistance); drought recovery, or the speed at which a tree recovers from a drought event (Recovery); drought resilience, or ability to achieve pre-drought growth (Resilience); and relative resilience, or the ability to regain pre-drought growth with respect to growth reduction severity during the drought event (Relative Resilience). Physiological variables include: hydraulic diameter in µm (Hydraulic Diameter); mean tracheid wall thickness in µm (Wall Thickness); stable oxygen isotope ratio ($\delta^{18}O$); stable carbon isotope ratio ($\delta^{13}C$); and intrinsic Water Use Efficiency, which is derived from the carbon isotope ratio (iWUE). Significance is indicated in bold and was adjusted using the Benjamini & Hochberg false discovery rate method.

Supplementary Table 4. Pearson's correlation coefficients of annual site climate to growth and physiology

	Growth																Physiology															
	Basal Area Increment (cm^2)				Height Increment (m)				Hydraulic Diameter (µm)				Tracheid Wall Thickness (µm)				$\delta^{18}O$ (‰)				$\delta^{13}C$ (‰)				iWUE ($\mu mol\ mol^{-1}$)							
	LE	CI	SI	FS	LE	CI	SI	FS	LE	CI	SI	FS	LE	CI	SI	FS	LE	CI	SI	FS	LE	CI	SI	FS	LE	CI	SI	FS				
Max. temp., spring	0.04	0.18	0.15	0.21	0.30	0.39	0.05	0.21	0.24	0.17	0.15	0.35	0.25	0.38	0.32	0.36	0.16	0.07	0.09	0.12	-0.23	-0.07	-0.05	-0.12	0.02	0.16	0.14	0.09				
Max. temp., summer	-0.20	-0.24	-0.19	-0.34	-0.25	-0.38	-0.22	-0.31	-0.05	-0.14	-0.18	-0.18	0.00	0.01	0.05	-0.02	**0.73**	**0.73**	**0.63**	**0.56**	0.39	**0.70**	**0.71**	**0.56**	**0.57**	**0.75**	**0.75**	**0.62**				
Max. temp., autumn	0.15	0.15	0.10	0.15	0.15	-0.03	-0.10	-0.17	-0.11	-0.03	-0.27	-0.18	0.09	0.03	0.05	0.08	-0.07	-0.16	-0.10	-0.04	0.02	0.09	0.12	0.23	0.08	0.12	0.15	0.24				
Precip. as snow	0.44	0.48	**0.53**	0.35	0.40	0.40	**0.58**	**0.50**	**0.56**	**0.51**	0.32	**0.54**	**0.43**	**0.42**	0.40	0.16	-0.25	-0.27	-0.10	-0.29	-0.18	-0.39	-0.09	-0.32	**-0.41**	**-0.54**	-0.25	**-0.46**				
Precip., spring	0.19	0.22	0.39	-0.05	0.17	0.14	**0.53**	0.15	**0.49**	**0.44**	0.28	0.30	**0.42**	**0.50**	0.42	0.10	0.24	0.21	0.31	0.05	-0.10	-0.06	0.27	-0.08	-0.16	-0.10	0.19	-0.12				
Precip., summer	0.15	0.29	0.30	0.22	**0.55**	**0.68**	0.34	0.38	0.21	0.19	0.21	0.35	0.23	**0.41**	0.22	0.11	**-0.46**	**-0.55**	-0.41	**-0.49**	**-0.59**	**-0.59**	**-0.55**	**-0.63**	**-0.59**	**-0.51**	**-0.48**	**-0.55**				
Precip., autumn	0.34	0.41	0.41	0.30	0.35	0.37	0.29	**0.55**	**0.54**	0.41	0.36	**0.62**	0.38	0.31	0.36	-0.01	0.21	0.20	0.29	0.10	0.01	0.02	0.19	-0.11	-0.07	-0.04	0.11	-0.15				
Rel. hum., spring	0.08	0.11	0.29	-0.20	0.20	0.10	**0.48**	0.11	0.20	0.35	0.30	0.09	0.09	0.18	0.07	-0.42	0.03	0.04	0.13	-0.15	-0.22	-0.11	0.10	-0.13	-0.37	-0.22	-0.02	-0.23				
Rel. hum., summer	0.23	0.39	0.42	0.29	**0.57**	**0.69**	**0.50**	0.41	0.25	0.36	0.31	0.38	0.22	0.41	0.20	0.05	**-0.74**	**-0.78**	**-0.61**	**-0.70**	**-0.69**	**-0.78**	**-0.68**	**-0.72**	**-0.79**	**-0.76**	**-0.67**	**-0.71**				
Rel. hum., autumn	0.13	0.33	0.41	0.15	**0.50**	**0.62**	**0.57**	**0.57**	**0.53**	**0.56**	**0.54**	**0.66**	0.30	**0.59**	0.40	0.15	-0.21	-0.22	-0.13	-0.33	**-0.54**	**-0.51**	-0.31	**-0.61**	**-0.56**	**-0.45**	-0.29	**-0.55**				

Climate variables include: maximum temperatures (Max. temp.) in spring, summer and autumn; precipitation (Precip.) as snow, in spring and in summer; and relative humidity (Rel. hum.) in spring, summer and autumn. Correlations of growth and physiology to climate are shown for the four tree populations representing the entire distribution of lodgepole pine (*Pinus contorta*). The four populations are represented by columns titled LE, CI, SI and TE: LE stands for the Leading Edge, i.e., the northern population occupying the area expected to be the leading edge of tree species migrations under climate warming; CI is Central Interior population, located in the central areas of the lodgepole pine distribution; SI is Southern Interior population, covering the mid-southern range of the lodgepole pine distribution; TE stands for the Trailing Edge, which represents seed sources from the far south of the lodgepole pine distribution, which is expected to see increased forest maladaptation under climate warming. Bold values indicate significant relationships, as adjusted using the Benjamini & Hochberg false discovery rate method.

Supplementary Table 5. Least square means and standard errors of growth, drought and isotope values

Regional Population	Growth		Physiology				
	Basal Area Increment (cm^2)	Height Increment (m)	Hydraulic Diameter (µm)	Mean Tracheid Wall Thickness (µm)	$\delta^{18}O$ (‰)	$\delta^{13}C$ (‰)	iWUE (µmol mol^{-1})
North (Leading Edge)	5.00 (0.15)	0.33 (0.01)	31.62 (0.19)	3.09 (0.03)	19.49 (0.06)	-26.77 (0.04)	79.74 (0.49)
Central Interior (Central to Distribution)	7.69 (0.15)	0.42 (0.01)	34.13 (0.19)	3.48 (0.03)	19.90 (0.06)	-26.23 (0.04)	85.54 (0.50)
Southern Interior (Central to Distribution)	8.53 (0.14)	0.42 (0.01)	34.64 (0.20)	3.44 (0.03)	20.08 (0.06)	-26.39 (0.04)	83.82 (0.48)
Far South (Trailing Edge)	6.31 (0.15)	0.38 (0.01)	31.74 (0.20)	3.38 (0.03)	19.88 (0.06)	-26.66 (0.05)	80.92 (0.51)

Least square means averaged over the 10-year study period for the four populations representing the full north-south range of interior lodgepole pine (*Pinus contorta* ssp. *latifolia*). Populations are represented by five provenances grouped into four climatic regions, LE, CI, SI and TE: LE stands for the Leading Edge, i.e. the northern population occupying the area expected to be the leading edge of tree species migrations under climate warming; CI is Central Interior population, located in the central areas of the lodgepole pine range; SI is the Southern Interior population covering the southern range of the central areas of the lodgepole pine range; TE stands for the Trailing Edge, which represents seed sources from the far south of the lodgepole pine range, which is expected to see increased forest maladaptation under climate warming. Intrinsic Water Use Efficiency (iWUE) is derived from tree-ring $\delta^{13}C$. Standard errors of the estimates are provided in brackets.

Supplementary Table 6. Multiple comparisons among populations' growth increments

Test	Basal area increment				Height increment			
	Est.	SE	z value	p-value	Est.	SE	z value	p-value
LE - CI == 0	-2.68	0.60	-4.47	**<0.001**	-0.10	0.02	-5.36	**<0.001**
SI - CI == 0	0.85	0.59	1.43	0.481	0.00	0.02	0.05	0.999
TE - CI == 0	-1.38	0.61	-2.26	0.108	-0.04	0.02	-2.27	0.104
SI - LE == 0	3.53	0.59	5.96	**<0.001**	0.10	0.02	5.55	**<0.001**
TE - LE == 0	1.30	0.61	2.13	0.144	0.05	0.02	3.04	**0.013**
TE - SI == 0	-2.23	0.60	-3.69	**0.001**	-0.04	0.02	-2.37	0.082

Multiple comparisons among tree populations representing the full north-south range of interior lodgepole pine (*Pinus contorta* ssp. *latifolia*). Populations are represented by five provenances grouped into four climatic regions, LE, CI, SI and TE: LE stands for the Leading Edge, i.e. the northern population occupying the area expected to be the leading edge of tree species migrations under climate warming; CI is Central Interior population, located in the central areas of the lodgepole pine range; SI is the Southern Interior population covering the southern range of the central areas of the lodgepole pine range; TE stands for the Trailing Edge, which represents seed sources from the far south of the lodgepole pine range, which is expected to see increased forest maladaptation under climate warming. Estimate is indicated by Est. and standard error is indicated by SE. Significance ($\alpha = 0.05$) is indicated in bold and p-values were adjusted with the Benjamini & Hochberg false discovery rate method.

Supplementary Table 7. Multiple comparisons among populations' drought tolerance indicators

Test	Resistance				Recovery				Resilience				Relative Resilience			
	Est.	SE	z value	p-value	Est.	SE	z value	p-value	Est.	SE	z value	p-value	Est.	SE	z value	p-value
LE - CI == 0	0.00	0.02	0.09	>0.999	-0.09	0.04	-2.18	0.130	-0.07	0.03	-2.13	0.145	-0.07	0.03	-2.72	**0.033**
SI - CI == 0	0.01	0.02	0.46	0.968	-0.01	0.04	-0.12	0.999	0.01	0.03	0.25	0.995	0.00	0.03	-0.11	>0.999
TE - CI == 0	-0.07	0.02	-2.98	**0.016**	0.14	0.04	3.38	**0.004**	0.00	0.03	0.00	>0.999	0.07	0.03	2.82	**0.025**
SI - LE == 0	0.01	0.02	0.37	0.983	0.09	0.04	2.03	0.176	0.08	0.03	2.35	0.087	0.07	0.03	2.58	**0.048**
TE - LE == 0	-0.08	0.02	-3.06	**0.012**	0.23	0.04	5.51	**<0.001**	0.07	0.03	2.09	0.156	0.14	0.03	5.49	**<0.001**
TE - SI == 0	-0.09	0.03	-3.40	**0.004**	0.15	0.04	3.47	**0.003**	-0.01	0.03	-0.24	0.995	0.08	0.03	2.91	**0.019**

Multiple comparisons among tree populations representing the full north-south range of interior lodgepole pine (*Pinus contorta* ssp. *latifolia*). Populations are represented by five provenances grouped into four climatic regions, LE, CI, SI and TE: LE stands for the Leading Edge, i.e. the northern population occupying the area expected to be the leading edge of tree species migrations under climate warming; CI is Central Interior population, located in the central areas of the lodgepole pine range; SI is the Southern Interior population covering the southern range of the central areas of the lodgepole pine range; TE stands for the Trailing Edge, which represents seed sources from the far south of the lodgepole pine range, which is expected to see increased forest maladaptation under climate warming. Estimate is indicated by Est. and standard error is indicated by SE. Significance (α = 0.05) is indicated in bold and p-values were adjusted with the Benjamini & Hochberg false discovery rate method.

Supplementary Table 8. Multiple comparisons among populations' tree-ring stable oxygen and carbon isotope values and derived intrinsic Water Use Efficiency (iWUE) values

Test	$\delta^{18}O$				$\delta^{13}C$				iWUE			
	Est.	SE	z value	p-value	Est.	SE	z value	p-value	Est.	SE	z value	p-value
LE - CI == 0	-0.41	0.15	-2.82	**0.025**	-0.54	0.14	-3.72	**0.001**	-5.80	1.55	-3.73	**0.001**
SI - CI == 0	0.19	0.14	1.29	0.570	-0.16	0.14	-1.13	0.674	-1.71	1.53	-1.12	0.678
TE - CI == 0	-0.01	0.15	-0.09	0.999	-0.43	0.15	-2.93	**0.018**	-4.62	1.58	-2.92	**0.018**
SI - LE == 0	0.60	0.14	4.18	**<0.001**	0.38	0.14	2.67	**0.038**	4.08	1.52	2.69	**0.036**
TE - LE == 0	0.40	0.15	2.71	**0.035**	0.11	0.15	0.74	0.882	1.18	1.57	0.75	0.876
TE - SI == 0	-0.20	0.15	-1.36	0.522	-0.27	0.14	-1.87	0.241	-2.90	1.55	-1.87	0.239

Multiple comparisons among tree populations representing the full north-south range of interior lodgepole pine (*Pinus contorta* ssp. *latifolia*). Populations are represented by five provenances grouped into four climatic regions, LE, CI, SI and TE: LE stands for the Leading Edge, i.e. the northern population occupying the area expected to be the leading edge of tree species migrations under climate warming; CI is Central Interior population, located in the central areas of the lodgepole pine range; SI is the Southern Interior population covering the southern range of the central areas of the lodgepole pine range; TE stands for the Trailing Edge, which represents seed sources from the far south of the lodgepole pine range, which is expected to see increased forest maladaptation under climate warming. Estimate is indicated by Est. and standard error is indicated by SE. Significance ($\alpha = 0.05$) is indicated in bold and p-values were adjusted with the Benjamini & Hochberg false discovery rate method.

Supplementary Table 9. Multiple comparisons among populations' functional wood anatomical properties

Test	Hydraulic mean diameter				Mean tracheid wall length			
	Est.	SE	z value	p-value	Est.	SE	z value	p-value
LE - CI == 0	-2.51	0.82	-3.07	**0.011**	-0.39	0.11	-3.61	**0.002**
SI - CI == 0	0.51	0.83	0.62	0.927	-0.04	0.11	-0.35	0.985
TE - CI == 0	-2.39	0.84	-2.84	**0.023**	-0.10	0.11	-0.89	0.811
SI - LE == 0	3.02	0.80	3.78	**<0.001**	0.35	0.11	3.32	**0.005**
TE - LE == 0	0.12	0.81	0.15	0.999	0.29	0.11	2.72	**0.033**
TE - SI == 0	-2.90	0.83	-3.51	**0.002**	-0.06	0.11	-0.55	0.948

Multiple comparisons among tree populations representing the full north-south range of interior lodgepole pine (*Pinus contorta* ssp. *latifolia*). Populations are represented by five provenances grouped into four climatic regions, LE, CI, SI and TE: LE stands for the Leading Edge, i.e. the northern population occupying the area expected to be the leading edge of tree species migrations under climate warming; CI is Central Interior population, located in the central areas of the lodgepole pine range; SI is the Southern Interior population covering the southern range of the central areas of the lodgepole pine range; TE stands for the Trailing Edge, which represents seed sources from the far south of the lodgepole pine range, which is expected to see increased forest maladaptation under climate warming. Estimate is indicated by Est. and standard error is indicated by SE. Significance ($\alpha = 0.05$) is indicated in bold and p-values were adjusted with the Benjamini & Hochberg false discovery rate method.

Supplementary Figures

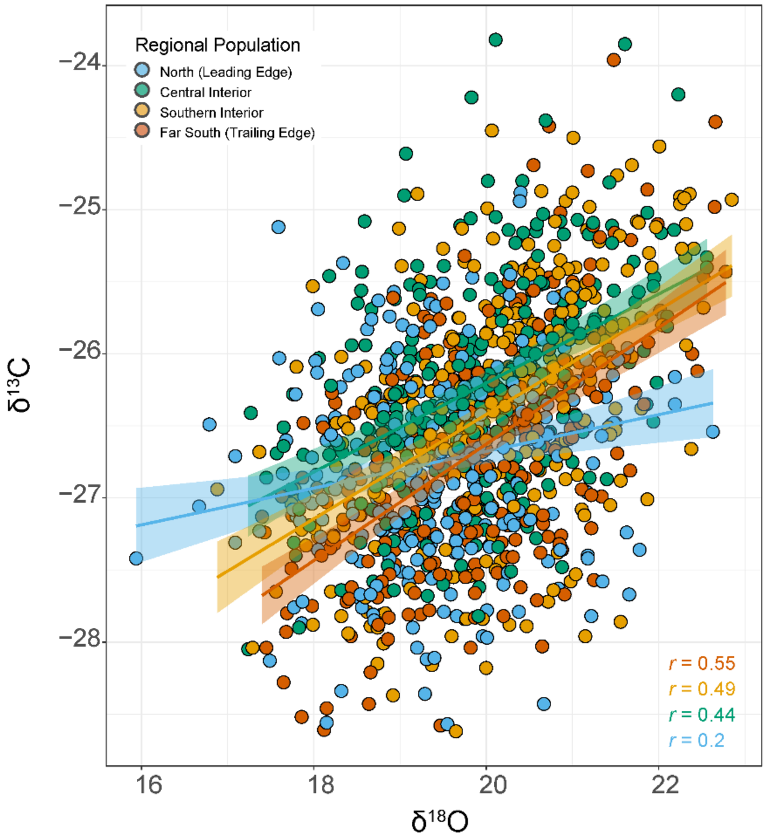

Supplementary Figure 1. Relationship between stable carbon and oxygen isotope ratios in four populations of lodgepole pine. Each dot represents the values from one tree (provenance) in one year at one block (n = 1170).

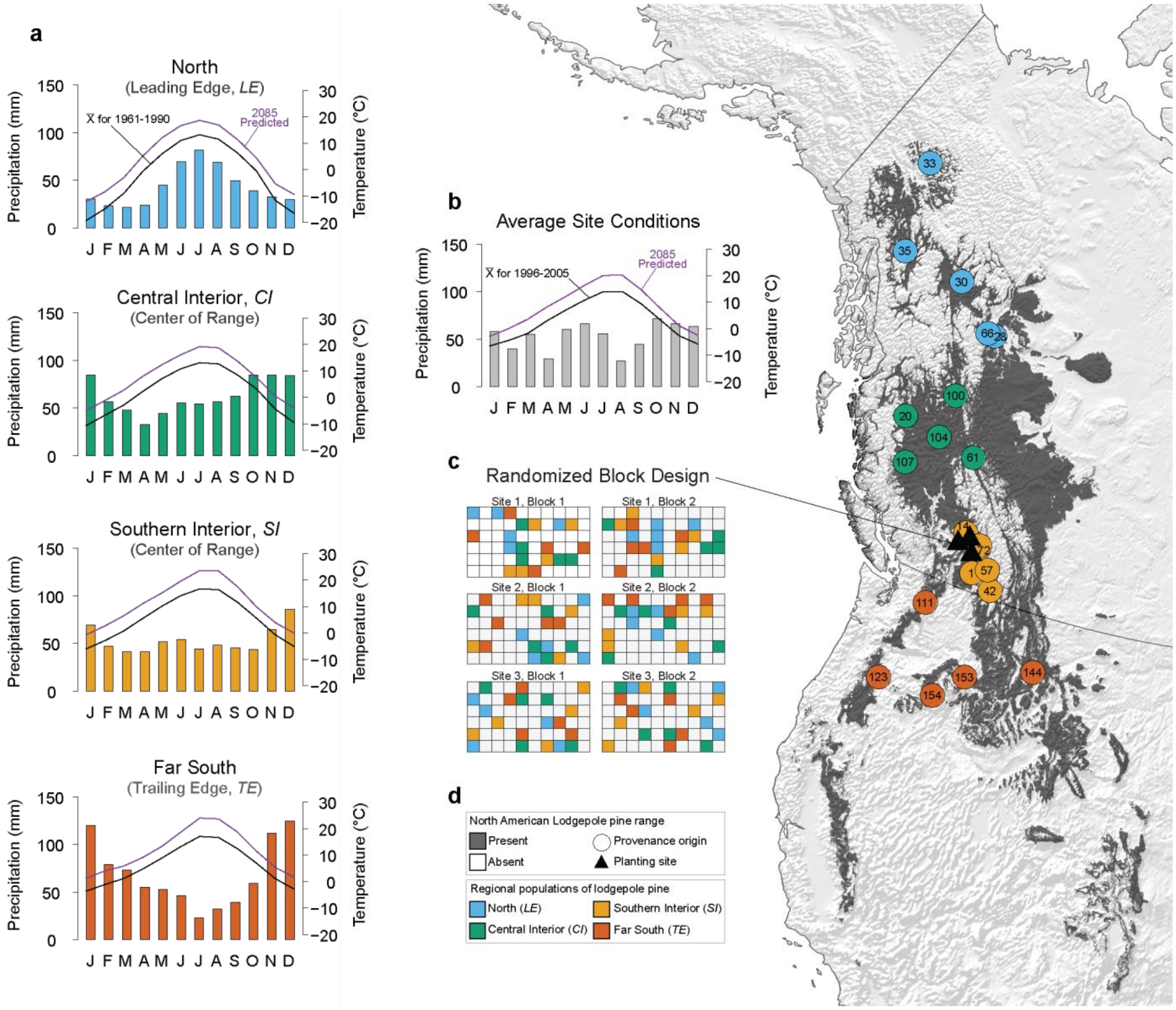

Supplementary Figure 2. Study design and climates across the species distribution of lodgepole pine (*Pinus contorta*). a) Climate graphs of precipitation (bars) and temperature (lines) for the average climates of the four regional populations b) Climate graph showing average conditions across the planting sites, where bars show precipitation and lines show temperature. c) The experiment relies on a complete randomized block design, where each provenance was planted in a randomized pattern across two blocks per site. d) Map and legend, where dark grey shows the lodgepole pine range, circles represent seed sources numbered according to the original provenance trial design by Illingworth and the three planting sites are depicted as black triangles. The map was produced by the authors with ArcInfo 10.1 using vector and raster data from www.naturalearthdata.com (Public Domain).

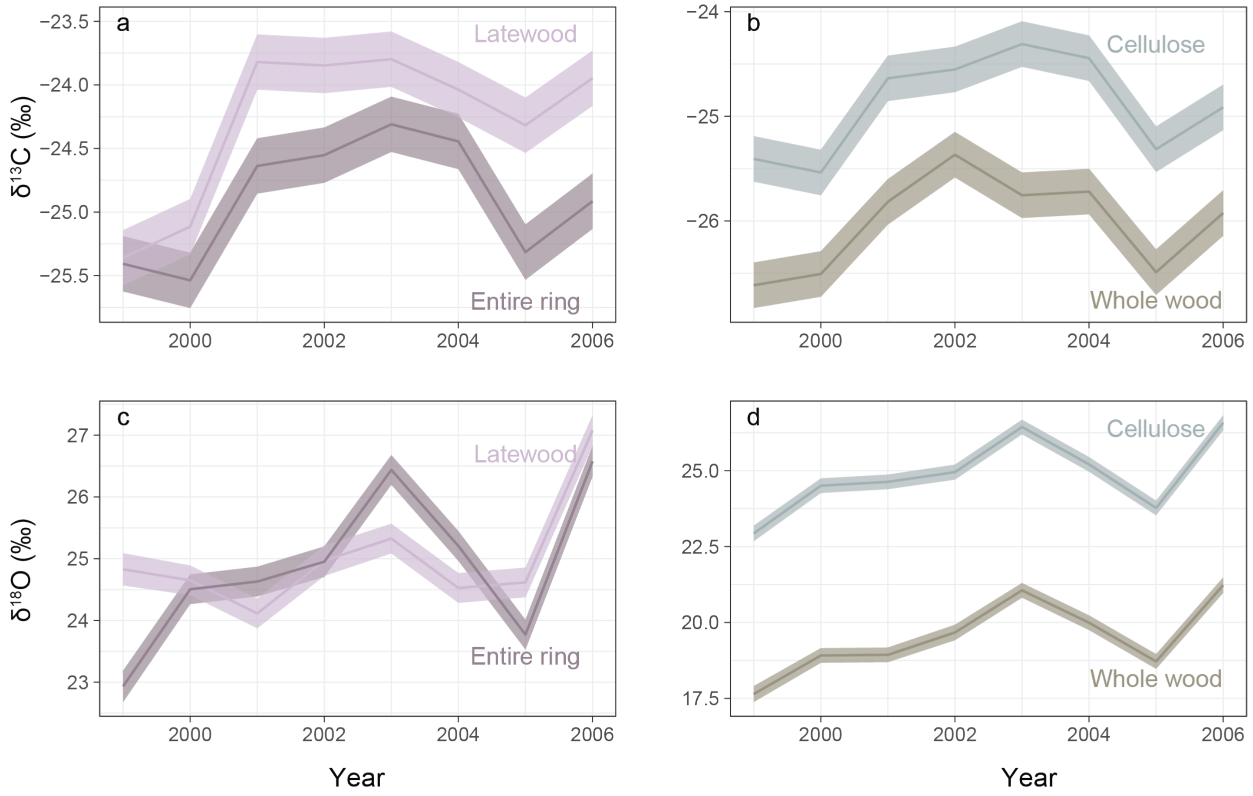

Supplementary Figure 3. Pilot study results comparing materials and methodologies for stable carbon and oxygen isotope analyses in tree rings from interior lodgepole pine (*Pinus contorta* ssp. *latifolia*). The two upper graphs correspond to stable carbon isotope ratios ($\delta^{13}C$) while the two lower graphs correspond to stable oxygen isotope ratios ($\delta^{18}O$). The two graphs on the left compare isotope signatures in the entire ring versus those in the latewood only. The two graphs on the right compare isotope signatures in cellulose versus those in resin-extracted whole-wood. The shaded areas represent standard errors of the mean.
